# Supplementary figures and images for: Identification of a Kingella kingae factor H binding protein that is the major determinant of serum resistance
Source: PLoS Pathog. 2025 Sep 2;21(9):e1013473. doi: 10.1371/journal.ppat.1013473 (PMC12416846; doi:10.1371/journal.ppat.1013473)

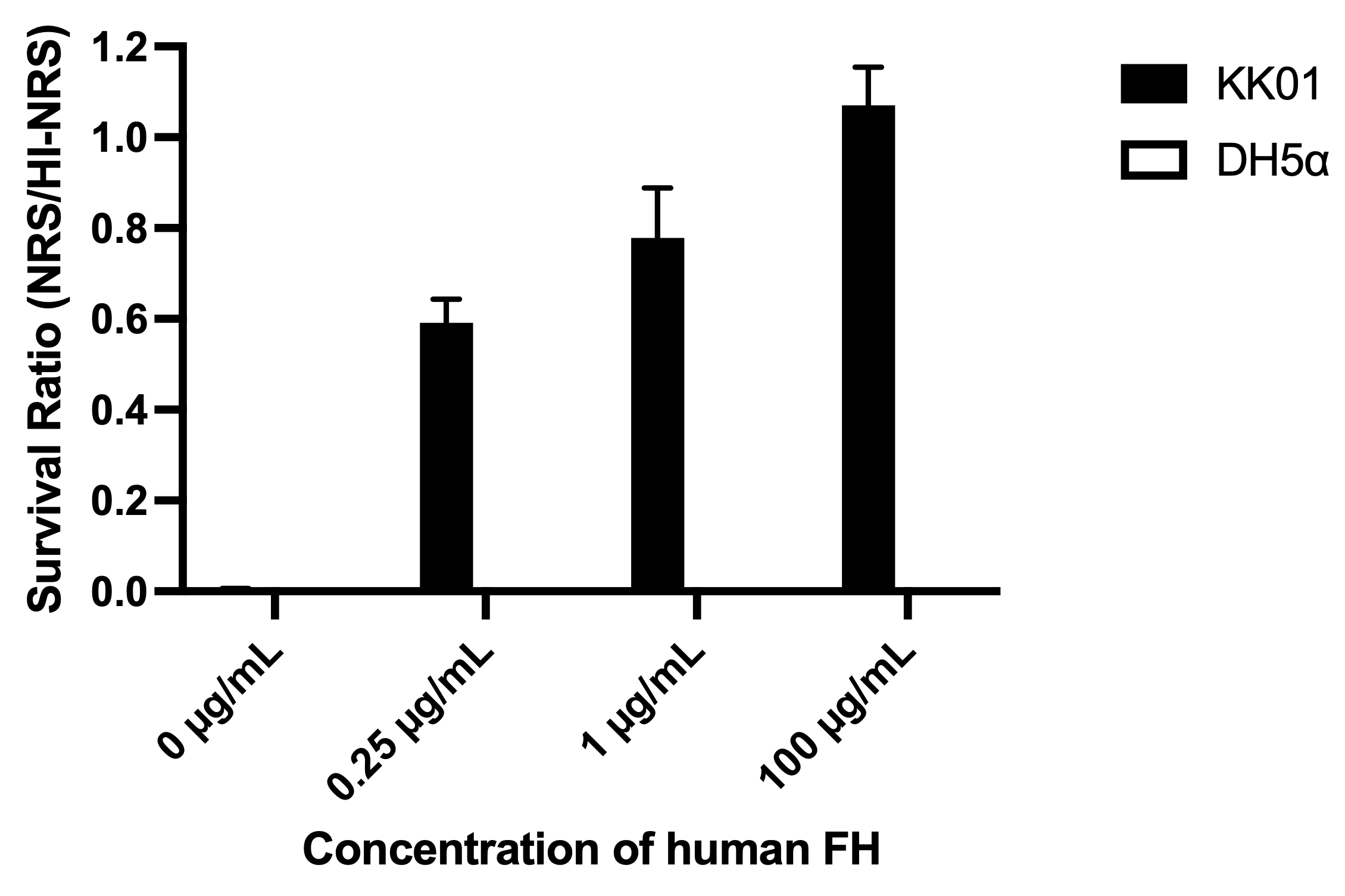

Supplement: S1 Fig — K. kingae strain KK01 and E. coli strain DH5α [~103 colony forming units (CFU)] were incubated with 5% normal rat serum (NRS) or heat-inactivated NRS (HI-NRS) with 0, 0.25, 1, or 100 µg/mL human FH. The survival ratio was calculated by dividing NRS CFU counts by the HI-NRS CFU counts. A total of 3 biological replicates were performed (n = 3). Data are presented as means, and the error bars represent the standard error of the mean. (TIFF) [file ppat.1013473.s001.tiff]

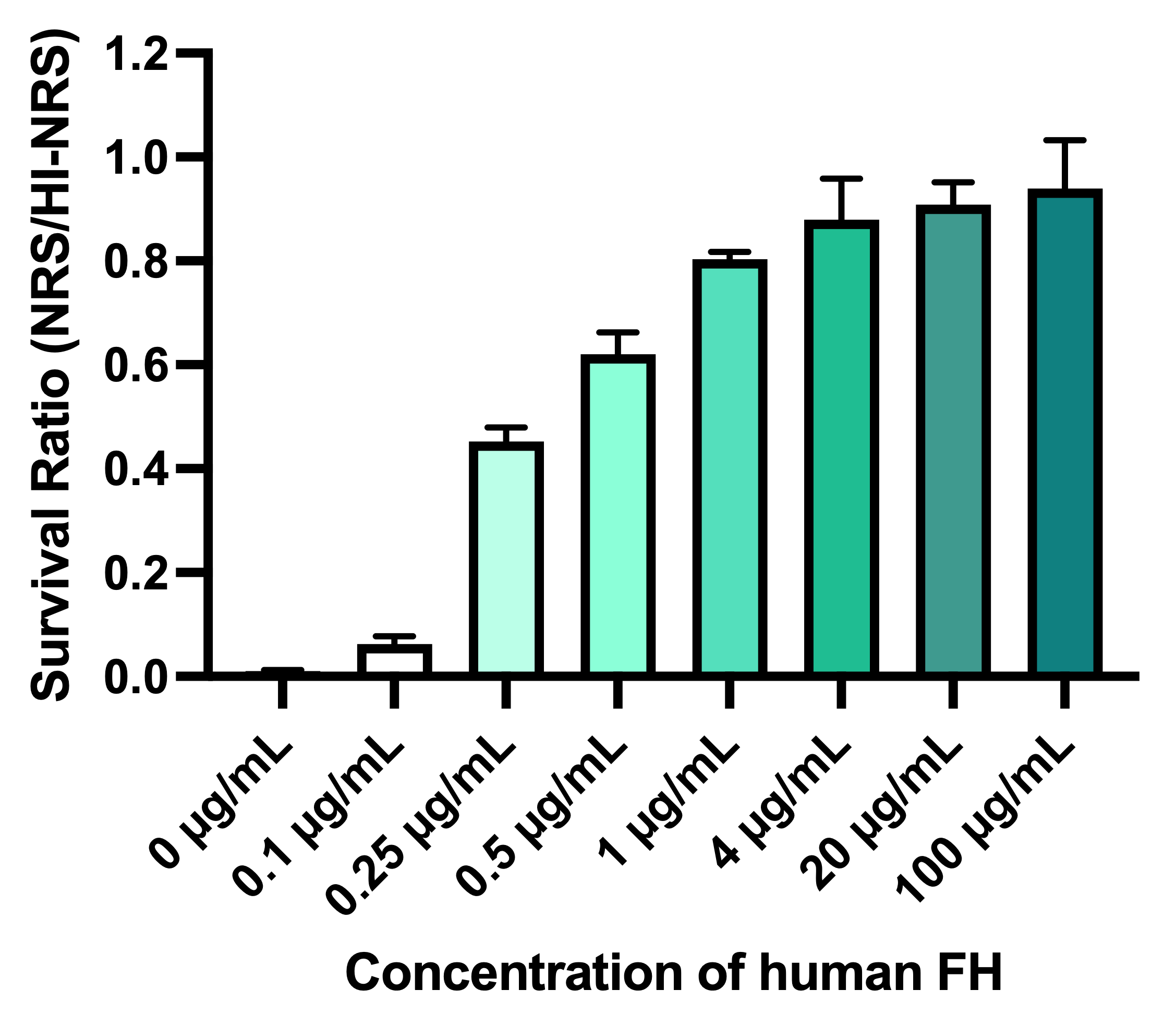

Supplement: S2 Fig — K. kingae strain KK01 (~103 CFU) was incubated with 5% NRS or HI-NRS with increasing concentrations of human FH, 0 µg/mL – 100 µg/mL. The survival ratio was calculated by dividing NRS CFU counts by the HI-NRS CFU counts. A total of 3 biological replicates were performed (n = 3). Data are presented as means, and the error bars represent the standard error of the mean. (TIFF) [file ppat.1013473.s002.tiff]

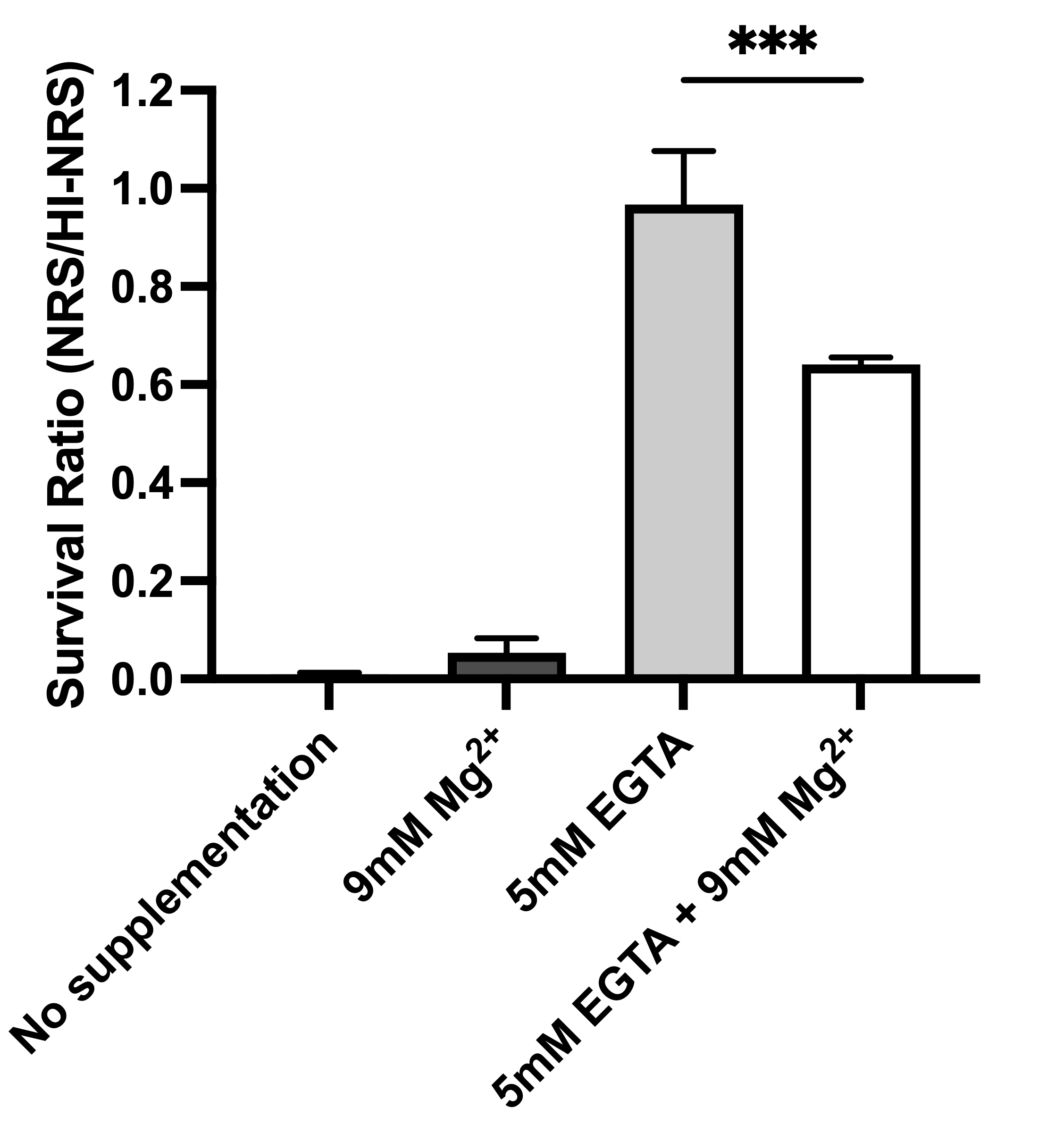

Supplement: S3 Fig — K. kingae strain KK01 (103 CFU) was incubated with either 5% NRS or 5% HI-NRS alone, 5% NRS or 5% HI-NRS plus 9mM Mg2+, 5% NRS or 5% HI-NRS plus EGTA, or 5% NRS or 5% HI-NRS plus EGTA and 9mM Mg2+. The survival ratio was calculated by dividing NRS CFU counts by the HI-NRS CFU counts. A total of 3 biological replicates were performed (n = 3). Data are presented as means, and the error bars represent the standard error of the mean. Statistical significance was determined by 1-way analysis of variance (ANOVA) with Tukey’s correction for multiple comparisons. ***, P < 0.001. (TIFF) [file ppat.1013473.s003.tiff]

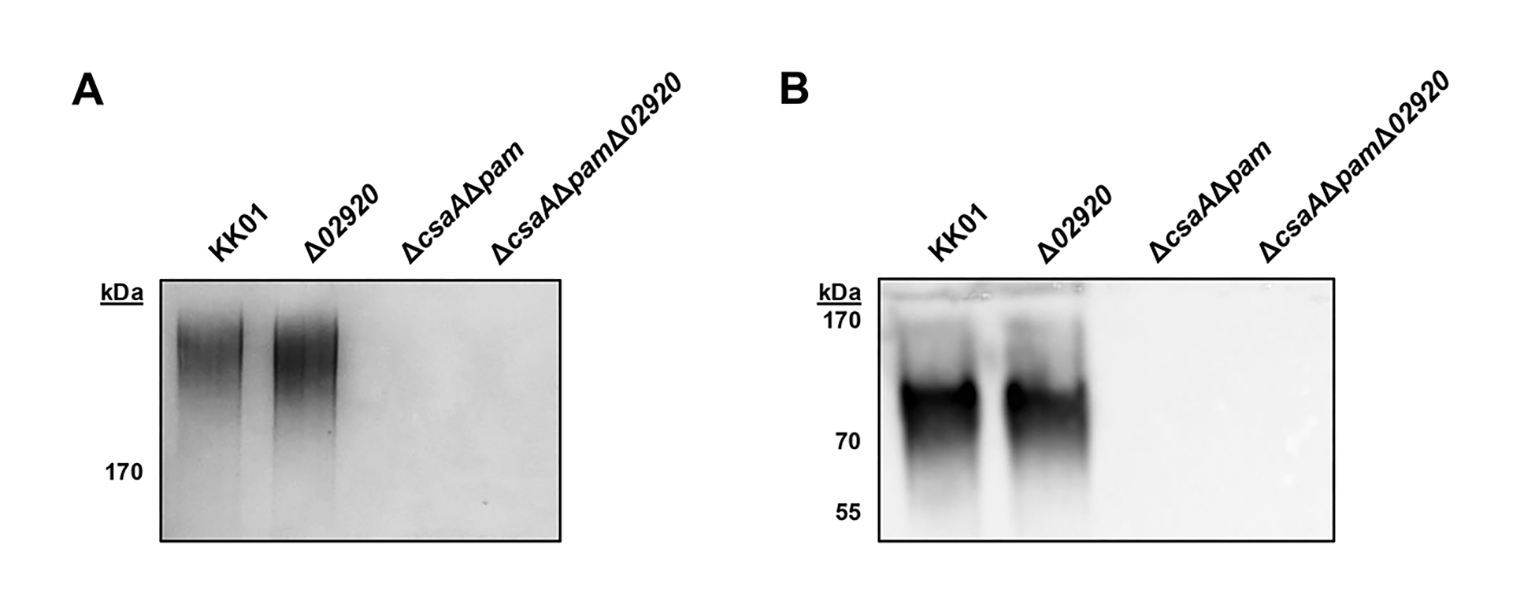

Supplement: S4 Fig — (A) Capsular material was extracted from K. kingae strains KK01 and KK01Δ02920, separated by 7.5% SDS-PAGE and then stained with the cationic dye alcian blue. K. kingae strains KK01ΔcsaAΔpam and KK01ΔcsaAΔpamΔ02920 were included as controls for no production of capsular material. The high molecular mass alcian blue-reactive material is indicative of the capsular material. A representative image is shown. (B) Galactan exopolysaccharide material was extracted from K. kingae strains KK01 and KK01Δ02920, separated by 16.5% DOC-PAGE, and transferred to a nitrocellulose membrane. A Western blot was performed by incubating the membrane with a galactan exopolysaccharide antiserum (GP-19) and anti-guinea pig HRP. The reactive mass spanning between 55 and 170 kDa is representative of the galactan exopolysaccharide. A representative image is shown. (TIF) [file ppat.1013473.s004.tif]
