# Supplementary material for: Identification of a Kingella kingae factor H binding protein that is the major determinant of serum resistance
Source: PLoS Pathog. 2025 Sep 2;21(9):e1013473. doi: 10.1371/journal.ppat.1013473 (PMC12416846; doi:10.1371/journal.ppat.1013473)
Supplement: Attachment — Submitted filename: FH_Binding_Manuscript_ResponsetoReviewersComments.docx [file ppat.1013473.s005.docx]

Dear Editors,

Below are our responses to the reviewer comments for manuscript submission PPATHOGENS-D-25-00712. We thank the reviewers for their careful review and their helpful suggestions, which have allowed us to improve the manuscript. Our responses are in **bold** and are directly below each reviewer comment.

Major issues

Reviewer #1:

The potential value of KK02920 as a component of a protective pediatric vaccine raises a few questions: *Kingella kingae* exhibits a wide range of genomic diversity, and the organism population comprises strains with remarkable differences in virulence. While some isolates are frequently carried on the oropharynx but are seldom if ever, recovered from invasive infections, a few others are responsible for the vast majority of osteoarthritis, bacteremia, and endocarditis cases. Following this line of thought:

1. What was the clinical source of the original (wild-type) KK01 strain (asymptomatic carriage?/invasive disease?).

**Table 1 indicates that strain KK01 is a naturally occurring nonspreading and noncorroding variant of septic arthritis clinical isolate 269-492. In the revised manuscript, we have added this information in lines 64-66, immediately following the first mention of strain KK01 in the Results section.**

2. It will be important to examine other *K. kingae* strains, invasive and mere respiratory colonizers, for the presence of KK02920. The potential significance of KK02920 as a target for such a vaccine will depend on its prevalence in the *K. kingae* population and, particularly, among strains of well-recognized invasiveness.

**In the revised manuscript, we have added a far-western blot of outer membrane preparations from a sample of carrier and invasive isolates from across the *K. kingae* population structure that have been previously determined to be resistant to the bactericidal effects of human serum (Muñoz et al. 2018 *Infect Immun*). As shown in Fig 9A, all isolates contain a band that is approximately the same size as KK02920 in strain KK01, suggesting that KK02920 is widespread across the *K. kingae* population structure. In future work we will undertake more detailed studies to determine the prevalence and conservation of KK02920 across a larger collection of *K. kingae* isolates.**

**We have added these clinical isolates to Table 1, and we have added text that addresses these results in the Results section (lines 259-266) and the Discussion section (lines 345-356).**

Reviewer #2: none

Reviewer #3:

1. The differentiation of which complement pathway is largely responsible for *K. kingae* killing was determined. Factor H is an alternative pathway regulator. The authors need to demonstrate that this killing is mediated via this pathway, not the other two. Standards assays are well-established in the field to make this differentiation. The previous study published by the group demonstrated that substantial killing is antibody-mediated (i.e. classical pathway).

**In response to this comment, we performed serum bactericidal assays using serum supplemented with EGTA and Mg^2+^ to selectively inhibit the classical and lectin pathways, thereby isolating the alternative pathway. As shown in Fig 7B in the revised manuscript, the addition of EGTA and Mg^2+^ to serum restored survival of strain KK01Δ*csaA*Δ*pam* to the level of strain KK01. In contrast, the addition of EGTA and Mg^2+^ to serum resulted in survival of strain KK01Δ*02920* that was reduced by ~80% compared to strain KK01, indicating that KK02920 affects resistance the alternative pathway. Similarly, the addition of EGTA and Mg^2+^ to serum resulted in survival of strain KK01Δ*csaA*Δ*pam*Δ*02920* that was reduced by >99% compared to strain KK01Δ*csaA*Δ*pam*, again indicating that KK02920 affects resistance the alternative pathway. Although nonsignificant (P = 0.19), the addition of EGTA and Mg^2+^ to serum resulted in survival of strain KK01Δ*csaA*Δ*pam*Δ*02920* that was reduced by ~20% compared to strain KK01Δ*02920*, indicating that the capsule and exopolysaccharide play a minor role in inhibiting resistance to the alternative pathway. These results suggest that KK02920 plays a critical role in protecting against the alternative pathway in serum killing, while the capsule and exopolysaccharide contribute modestly to resistance to the alternative pathway.**

**In the revised manuscript, we have also modified the Methods (lines 466-468), the Results (lines 226-239), and the Discussion (lines 323-332) to reflect these new findings.**

2. The authors demonstrated the binding of fH. Still, they did not demonstrate that this bound fH is functional (Cofactor Activity for Factor I cleaving C3b and Decay-Accelerating Activity displacing Bb from the C3 convertase). Standard assays are well-established in the field to prove this functionality.

**In response to this comment, we performed a cofactor assay to demonstrate that bound FH to *K. kingae* is functional in serving as a cofactor for Factor I (FI) and cleaving C3b. Strains KK01Δ*csaA*Δ*pam* and KK01Δ*csaA*Δ*pam*Δ*02920* were incubated with FH and were then incubated with C3b and FI and resolved by SDS-PAGE. As controls, we incubated bacteria in the absence of FH or FI. As demonstrated in Fig 8 in the revised manuscript, the C3b degradation fragments α′ 43-kDa and α′ 41-kDa were absent in samples lacking FH or FI, indicating that both of these factors are necessary for C3b degradation. Incubation of strain KK01Δ*csaA*Δ*pam* with FH and FI produced the α′ 43-kDa and α′ 41-kDa C3b degradation fragments. In contrast, these degradation fragments were markedly reduced when incubating strain KK01Δ*csaA*Δ*pam*Δ*02920* with FI and FI. These results indicate that FH retains cofactor activity when bound to *K. kingae* and is dependent on KK02920.**

**In the revised manuscript, we have also modified the Methods (lines 575-592), the Results (lines 241-255), and the Discussion (lines 326-329) to reflect these new findings.**

3. For the flow cytometry experiments, could the author provide representative histograms of the obtained results, especially for the experiments described in Figure 1?

**In the revised manuscript, we have modified Figure 1 to include representative histograms (now Fig 1A) along with the bar graph depicting the quantified results (now Fig 1B). In addition, we have modified Figure 5 to include representative histograms (now Fig 5A) along with the bar graph depicting the quantified results (now Fig 5B).**

4. In Figure 2B, what is considered the 100% survival?

**In the 100% survival condition (survival ratio of 1.0), the number of bacteria that survived treatment with normal rat serum is equivalent to the number of bacteria that survived treatment with heat-inactivated normal rat serum.**

5. What is the identified target protein conservation among different K. kingae strains?

**Please see our explanation in the response to “Major issue” 2 from reviewer #1.**

6. The binding and protection need to be tested among a diverse panel of K. kingae strains to demonstrate the generalizability of the observed phenotypes.

**Please see our explanation in the response to “Major issue” 2 from reviewer #1.**

Minor issues:

Reviewer #1: No response

Reviewer: #2: This paper shows that *K. kingae* can bind human FH through a protein called KK02920. Human FH can restore virulence of *K. kingae* in infant rats. The experiments are rigorous, and the results are presented clearly. Minor comments, mostly editorial, are presented below for the authors’ consideration.

1. Suggest using FH instead of fH to be consistent with the recommendations of the International Complement Society (PMID: 31231398).

**In the revised manuscript, we have changed fH to FH.**

2. Line 17-20: May be worth also mentioning that the AP can also be triggered by C3b deposited through the CP and LP. In fact, spontaneous activation (alone) is very inefficient (PMID: 36424888).

**We appreciate this suggestion. In the revised manuscript, we have added the following text in line 19-22 in the Introduction:**

**“However, recent studies have shown that spontaneous complement deposition may be inefficient and that the alternative pathway may serve primarily as an amplification mechanism, triggered by other means such as the classical and lectin pathway.”**

3. The authors should provide representative flow cytometry tracings or data shown in Fig. 1.

**Please see our explanation in response to “Major issue” 3 from reviewer #3.**

4. If mAb OX-24 was used to detect FH binding when bacteria or western blot membranes were incubated with human serum (HI-NHS), please state either FH and/or the alternatively spliced molecule, FHL-1 could have been detected (PMID: 2936333). But Fig. 4 with pure FH suggests unequivocally that full length FH can bind to KK02920, as do the ‘rescue’ experiments with pure FH in juvenile rats.

**In the revised manuscript, we have clarified in the Results section (lines 69-71) that mAB OX-24 detects both FH and FHL-1. We have revised the description of the flow cytometry data using HI-NHS (Fig 1) to specify that the observed binding reflects the presence of FH and/or FHL-1, rather than FH alone. We have also updated the figure title and figure legend.**

**Subsequent in vitro and in vivo protection assays (Figs 2 & 3) and the initial far-western blot (Fig 4) were performed using purified human FH, confirming that *K. kingae* binds FH.**

5. What was the age of the rats from which rat serum was prepared (lines 371-372)? It is remarkable that even 0.25 mcg/mL of human FH can rescue the bacteria from killing by rat serum, which would not be expected if killing was driven by the classical pathway.

**The age of the rats was approximately 3 to 5 months.**

6. In light if the comment above, it may be worth performing serum bactericidal assays in NHS and NRS with the csaA/pam KO strains where only the AP is active – for eg, using Mg-EGTA-treated serum.

**Please see our explanation in response to “Major issue” 1 from reviewer #3.**

**To address whether rat serum (NRS) killing in the absence of human FH is driven by the alternative pathway, we performed a serum bactericidal assay with 5% NRS supplemented with EGTA and Mg^2+^. As mentioned in response to “Major issue” 1 from reviewer #3, EGTA chelates Ca^2+^ and thereby blocks activation of the classical pathway, while the concomitant addition of Mg^2+^ maintains the alternative pathway. As shown in Fig S3 in the revised manuscript, the addition of EGTA to NRS virtually fully restored serum survival of strain KK01, while the addition of EGTA plus Mg^2+^ restored survival to a survival ratio of about 0.65. These results indicate that NRS has classical and alternative pathway activity, contributing to NRS killing. In the revised manuscript, we have updated the Methods section (lines 466-468) and have added text in the Results section (lines 115-125).**

Reviewer #3

1. Gram should be capitalized throughout the manuscript.

**In the revised manuscript, we capitalized Gram.**

2. In vivo should not be italicized.

**In the revised manuscript, we have removed the italicization of in vivo.**

3. Western should not be capitalized.

**In the revised manuscript, we have changed Western to be all lowercase.**

4. The prime sign should be used instead of the apostrophe sign.

**In the revised manuscript, we have replaced the apostrophe sign with a prime sign where appropriate.**

5. Bacterial species names should be italicized, for example, on line 449.

**In the revised manuscript, we have italicized all bacterial species names.**

6. Please be consistent; sometimes, a space is left between the numerical value and the unit of measurement, and in others, no space is left.

**We have addressed this issue throughout the revised manuscript.**

We hope that the modified manuscript is now acceptable for publication in *PLOS Pathogens*. Please let me know if you have any further questions.

Sincerely,
Joe St. Geme
